# Surgical Treatment of Pediatric Incidentally Found Brain Tumors: A Single-Center Experience

**DOI:** 10.3390/brainsci13050746

**Published:** 2023-04-29

**Authors:** Lukasz Antkowiak, Mikolaj Zimny, Krzysztof Starszak, Ryszard Sordyl, Marek Mandera

**Affiliations:** 1 Department of Pediatric Neurosurgery, Medical University of Silesia in Katowice, 40-752 Katowice, Poland; krzysztof.starszak@gmail.com (K.S.); ryszard.sordyl@sum.edu.pl (R.S.); mmandera@sum.edu.pl (M.M.); 2 Department of Neurosurgery, Medical University of Silesia in Katowice, 40-752 Katowice, Poland; zimny.mikolaj@gmail.com; 3 Department of Human Anatomy, Medical University of Silesia in Katowice, 40-752 Katowice, Poland

**Keywords:** incidentaloma, brain tumor, incidental, pediatric, management, low-grade glioma, oncology

## Abstract

There remains much debate about the correct management of incidentally found brain tumors in the pediatric population. This study aimed to evaluate the efficacy and safety of surgical treatment of incidentally found pediatric brain tumors. A retrospective analysis of pediatric patients who underwent surgical resection of incidentally found brain tumors between January 2010 and April 2016 was performed. A total of seven patients were included. The median age at the time of diagnosis was 9.7 years. The reasons for performing neuroimaging were as follows: impeded speech development (*n* = 2), shunt control (*n* = 1), paranasal sinuses control (*n* = 1), behavior changes (*n* = 1), head trauma (*n* = 1), and preterm birth (*n* = 1). Five patients underwent gross total tumor resection (71.4%), while subtotal resection was performed in two patients (28.6%). There was no surgery-related morbidity. Patients were followed up for a mean of 79 months. One patient with atypical neurocytoma experienced tumor recurrence 45 months following primary resection. All patients remained neurologically intact. The majority of pediatric incidentally found brain tumors were histologically benign. Surgery remains a safe therapeutic approach associated with favorable long-term outcomes. Considering the expected long lifetime of pediatric patients, as well as the psychological burden associated with having a brain tumor as a child, surgical resection can be considered an initial approach.

## 1. Introduction

The constantly growing availability of neuroimaging has caused an increase in incidental intracranial findings. Such findings are found in 21–25% of children undergoing brain imaging [1,2]. Incidentally found brain tumors, also referred to as incidentalomas, account for 0.2–5.7% of all incidental intracranial findings, with an estimated prevalence of 0.04–0.18% [1,3,4]. These lesions are defined as asymptomatic intracranial space-occupying lesions, and their radiological appearance is highly indicative of brain tumors [5]. The majority of pediatric incidentalomas are low-grade gliomas (LGG) [6]. While adult LGGs are at risk of malignant transformation (MT), pediatric LGGs rarely undergo MT. However, MT can still occur in up to 6.7% of pediatric patients with LGGs [6,7]. 

There is disagreement about whether it is best to apply a conservative “wait and scan” strategy with repeated MRI control or invasively intervene, either with biopsy or surgical tumor excision, aimed at gross-total resection (GTR) of the tumor. While no consensus regarding the optimal strategy has been reached, an individually tailored approach needs to be applied [8]. Due to the longer expected lifetime of children and the lower risk of MT of pediatric tumors, the management of incidentalomas in children should be different to that of incidentalomas in adults. However, since the diagnosis of an intracranial space-occupying lesion that is highly suspected to indicate a brain tumor produces a significant psychological burden for the patient and their family; the decision-making process regarding treatment should involve the active participation of both the patient and their family, including their concerns and preferences. The recent literature suggests a strategy of initial surveillance, with surgical intervention applied as the tumor progresses or as symptoms or signs of malignancy emerge [9]. 

Notably, there are no definitive radiologic criteria differentiating LGGs from malignant lesions, rendering the accuracy of radiologic surveillance debatable. Typically, the presence of a T1 hypointense and T2 hyperintense non-enhancing lesion without surrounding edema that does not produce a mass effect is reflective of a benign brain tumor [3,4]. Additionally, low or absent tracer uptake on positron emission tomography (PET) was suggested to be helpful in differentiating low-grade brain tumors from their malignant counterparts [10,11]. Magnetic resonance spectroscopy (MRS) is also beneficial for discriminating between non-neoplastic lesions or low-grade and high-grade tumors [12,13]. While it has already been suggested that MRS outperforms conventional MRI in brain tumor differentiation, the lack of prospective studies on pediatric incidentalomas prevents us from reaching a definitive conclusion on the applicability of MRS to the pediatric population [14]. 

Moreover, the lack of high-quality prospective studies involving long-term follow-up and a significant diversity between neurosurgical institutions regarding the preferred strategy means that the optimal management for children with incidentalomas remains controversial and institution dependent. The lack of guidelines on the management of pediatric incidentally found brain tumors together with the psychological burden associated with a suspected brain tumor in a child, the perioperative risk, and lack of surveillance standards render the decision-making process in children extremely difficult. Since the literature on the management of pediatric incidentally found brain tumors remains sparse, we aimed to evaluate the efficacy and safety of the initial surgical treatment of incidentally found brain tumors in the pediatric population.

## 2. Materials and Methods

### 2.1. Study Design

Following Ethical Committee approval (approval number BNW/NWN/0052/KB/62/23), we retrospectively analyzed the medical charts of pediatric patients who underwent surgical excision of incidentally found brain tumors between January 2010 and April 2016 in the Department of Pediatric Neurosurgery, Medical University of Silesia in Katowice. 

### 2.2. Inclusion and Exclusion Criteria

The present study focused only on pediatric patients (age at diagnosis < 18 years) treated with initial surgical excision of a space-occupying incidental mass within the brain. An incidental finding, also referred to as an incidentaloma, was defined as a space-occupying lesion found on brain-imaging studies performed for an unrelated condition (e.g., trauma or behavior changes) with a radiological appearance suggestive of a tumor in an asymptomatic patient [5,15]. Patients who were initially treated surgically (without a prior conservative approach) with complete clinical data and at least 12 months of postoperative follow-up were included in the study. Conversely, individuals with conditions known to predispose one to brain tumors (e.g., neurofibromatosis type I and II, Li Fraumeni syndrome, Von Hippel Lindau syndrome, and tuberous sclerosis) and individuals with radiological findings suggestive of demyelinating diseases (e.g., multiple sclerosis or acute sclerotic encephalomyelitis) or non-neoplastic lesions (e.g., Chiari malformation type I, arachnoid cyst, and vascular malformations) were excluded from the study.

### 2.3. Criteria Qualifying for Surgery

Since no definitive guidelines on the management of pediatric brain tumors exist, the decision-making process was made by an experienced pediatric neurosurgeon (M.M.) and remained individually tailored to each patient based on their imaging evaluation and parental preferences. Typically, the patient was considered eligible for surgical removal of the space-occupying mass if the following conditions were met: (1) a prominent mass effect was observed, (2) lesion growth was seen in the consecutive MRI examinations, and (3) imaging characteristics were suggestive of a neoplastic lesion. 

### 2.4. Data Extraction

Data were extracted on patients’ demographics, the reason for neuroimaging, lesion location, surgical procedure, the extent of resection (EOR), histopathology, postoperative clinical course, and clinical and radiological follow-up. 

## 3. Results

### 3.1. Patient Characteristics

A total of seven pediatric patients with incidentally found brain tumors were identified. The mean age at the time of diagnosis was 9.7 years (ranging from 21 months to 17 years). The reasons for undertaking neuroimaging included the following: impeded speech development (*n* = 2, 28.6%), shunt control (*n* = 1, 14.3%), paranasal sinuses control (*n* = 1, 14.3%), behavior changes (*n* = 1, 14.3%), head trauma (*n* = 1, 14.3%), and preterm birth (*n* =1, 14.3%). Six lesions (85.7%) were supratentorial, while the remaining lesion (14.3%) was infratentorial. Tumors were located within the lateral ventricle (*n* = 2, 28.6%), temporal lobe (*n* = 1, 14.3%), frontal lobe (*n* = 1, 14.3%), thalamus (*n* = 1, 14.3%), cerebellum (*n* = 1, 14.3%), and suprasellar region (*n* = 1, 14.3%). The patients’ characteristics are presented in Table 1.

### 3.2. Surgery and Histopathology

All patients qualified for surgical resection based on radiological criteria. The surgical intervention aimed for GTR in all patients whenever safe and technically feasible. No biopsy was performed before surgical excision in any patient. Five patients (71.4%) underwent GTR, while subtotal resection was carried out in two patients (28.6%), with one having dermoid cyst (the cyst wall was left due to the strict adherence to the sigmoid sinus) and the other had thalamic ganglioglioma (proximity to the eloquent brain area precluded safe GTR). 

Postoperative pathological examination revealed pilocytic astrocytoma (*n* = 2, 28.6%), choroid plexus papilloma (*n* = 1, 14.3%), ganglioglioma (*n* = 1, 14.3%), atypical neurocytoma (*n* = 1, 14.3%), dermoid cyst (*n* = 1, 14.3%), and craniopharyngioma (*n* = 1, 14.3%).

### 3.3. Clinical Evaluation

No intraoperative complications were observed. Postoperatively, patient no. 7 experienced wound dehiscence with cerebrospinal fluid (CSF) leakage. The wound was surgically managed without any additional sequelae. There was no postoperative neurological function deterioration in any patient. On discharge from the hospital, all patients remained asymptomatic and neurologically intact. 

Patients were followed up for the mean time of 79 months (ranging from 12 to 114 months). One patent initially treated for lateral ventricle atypical neurocytoma ((WHO (World Health Organization) grade 2 tumor)) experienced asymptomatic tumor recurrence after 45 months following primary GTR. Among the remaining six patients (85.7%), with all being followed up radiologically, no tumor recurrence was observed. No patient presented neurological decline within the follow-up period. 

Finally, four patients (57.1%) remained asymptomatic. One patient complained of sporadic headaches (patient no. 1), one of periodic syncope (patient no. 4), and the remaining patient presented signs of precocious puberty (patient no. 5). Due to the lack of a direct time correlation between the neurosurgical procedure and the later appearance of headaches in patient no. 1, the headaches were considered unrelated to the surgical tumor removal. Similarly, periodic syncope in the 14-year-old patient was considered to more likely result from the maturation process than the tumor removal procedure since neither a direct time correlation with the surgery nor a possible underlying mechanism of iatrogenic injury were identified. 

In contrast, patient no. 5, who underwent craniopharyngioma resection, presented signs of precocious puberty in the later postoperative period. It is unknown whether the patient presented hormonal equivalents of precocious puberty at diagnosis. We presume that the precocious puberty could have been caused either by the tumor itself, and that it remained undiagnosed preoperatively or by iatrogenic injury during surgery.

### 3.4. Case 1

**Figure 1:**
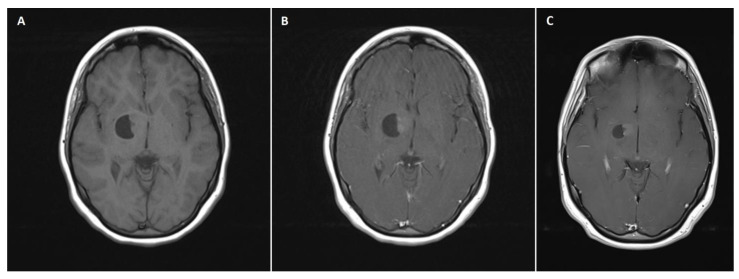
A 14-year-old girl with right-sided thalamic ganglioglioma. (**A**) A preoperative axial non-contrast T1 MRI scan showing an isodose solid mass located within the right thalamus with an adherent cystic component, causing a mass effect and slight midline shift. (**B**) The preoperative axial postcontrast T1 MRI scan revealed a heterogeneous solid mass enhancement. (**C**) A postoperative axial postcontrast T1 MRI scan reflecting subtotal excision of the tumor, with reduced size of the cystic component, relieved mass effect, and no evident midline shift.

A 14-year-old female patient with recently diagnosed pertussis underwent a CT scan of her paranasal sinuses. Imaging data revealed a left-sided space-occupying lesion at the level of the thalamus. The patient underwent a subsequent brain MRI scan, which showed a cystic–solid 3 cm × 2.5 cm mass located within the anterior thalamus and basal ganglia on the left side (Figure 1A,B). On admission, the patient was neurologically intact. Due to the mass effect exerted by the tumor, the patient qualified for surgical tumor removal. Considering the eloquent location of the adjacent neural tissue, GTR was not achieved to preserve neurological function postoperatively. No surgery-related complications occurred. A postoperative MRI scan confirmed subtotal resection of the tumor (Figure 1C). The patient was discharged home without any neurological deficits. Subsequent pathological examination revealed ganglioglioma (WHO grade 1 tumor). The patient has been followed up for 111 months, with neither radiological regrowth nor the occurrence of focal neurological symptoms. However, the patient complained of periodic headaches, which are unlikely to be caused by the surgery.

### 3.5. Case 2

An 11-year-old male patient with a ventriculoperitoneal shunt (VPS) implanted at the age of 3 months due to posthemorrhagic hydrocephalus underwent routine imaging control to evaluate the shunt function. A CT scan revealed a cystic lesion within the posterior part of the frontal lobe on the right side. A subsequently performed MRI scan confirmed a cystic 5 cm mass with an enhancing mural nodule. On admission, the patient was neurologically intact, without signs of raised intracranial pressure (ICP). Due to the compression of the right lateral ventricle, the patient qualified for surgical tumor excision. GTR was successfully achieved without compromising the patient’s neurological function. In the early postoperative period, the patient presented a tendency towards bradycardia, with sporadic nausea, vomiting, and headaches. A control CT scan revealed slightly dilated ventricles without signs of raised ICP. The patient was discharged from the hospital neurologically intact. Pathological examination revealed pilocytic astrocytoma (WHO grade 1 tumor). The patient was followed up for 74 months, being asymptomatic with no concurrent radiological signs of recurrence. 

### 3.6. Case 3

A 15-year-old left-handed male patient experienced head trauma due to a bicycle accident. Due to malaise and nausea, the patient underwent a head CT scan. Imaging examination did not reveal any post-traumatic intracranial abnormalities. However, a hypodense mass lesion within the left temporal lobe was observed. Further brain MRI scans confirmed a pathologic mass lesion. On admission, the patient was neurologically intact. The patient underwent surgical tumor excision. GTR of the tumor was achieved. A control head CT scan confirmed a normal postoperative state. The patient was discharged in an asymptomatic condition. Further pathological examination showed pilocytic astrocytoma (WHO grade 1 tumor). The patient was followed up for 92 months postoperatively. No signs of tumor recurrence were observed on imaging. Concurrently, the patient remained clinically intact.

### 3.7. Case 4

**Figure 2:**
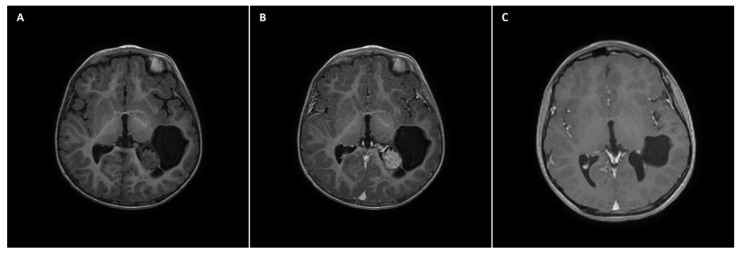
A 21-month-old boy with choroid plexus papilloma of the left lateral ventricle. (**A**) A preoperative axial non-contrast T1 MRI scan with a hypodense mass located within the left occipital horn of the lateral ventricle with cystic components. (**B**) The preoperative axial postcontrast T1 MRI scan showed homogeneous intraventricular mass enhancement, reflective of choroid plexus papilloma. (**C**) A postoperative axial postcontrast T1 MRI scan reflecting complete excision of the intraventricular mass, with the remaining cystic component.

A 21-month-old boy with a left lateral ventricle choroid plexus cyst diagnosed by means of USG in the neonatal period underwent CT and subsequent MRI due to progressive cyst enlargement. Imaging studies revealed a tumor of the choroid plexus within the left collateral triangle (Figure 2A,B). At admission, the patient was neurologically intact, with normal psychomotor development and no signs of raised ICP. Physical examination revealed slight cranial asymmetry, with bulging of the left parietal bone. The patient qualified for surgical tumor removal based on progressive enlargement of the pathologic mass. GTR of the tumor was achieved. In the early postoperative course, a few episodes of vomiting with raised body temperature occurred, but the patient remained neurologically intact. No signs of infection were detected in the blood tests. A postoperative head MRI scan confirmed complete tumor excision (Figure 2C). The patient was discharged home in an asymptomatic clinical state. Pathological examination revealed choroid plexus papilloma (WHO grade 1 tumor). The patient was followed up for 86 months with no radiological signs of tumor recurrence. Concurrently, episodes of sporadic syncope occurred clinically, but due to the lack of direct relation with surgical intervention, they were not considered to be associated with the tumor removal procedure. 

### 3.8. Case 5

A 3-year-old girl with impeded speech development underwent a brain MRI. Imaging data showed a cystic 7 mm suprasellar lesion. On admission, the patient was neurologically intact, but motor hyperactivity was observed. Eye examination was normal. Due to the high suspicion of craniopharyngioma, the patient qualified for surgical tumor removal. GTR was achieved without any surgery-related complications. Postoperatively, no signs of diabetes insipidus were observed. The control head CT scan showed a normal postoperative appearance within the operation site, with minor hygroma at the site of the performed craniotomy. The patient was discharged from the hospital without any neurological deficits. Pathological examination showed craniopharyngioma (WHO grade 1 tumor). The follow-up lasted for 65 months. No radiological signs of tumor recurrence were observed. The patient remained neurologically intact but presented signs of preterm maturation.

### 3.9. Case 6

A 6-year-old female patient with an impeded speech development underwent a brain MRI. Imaging revealed space-occupying mass lesions suggestive of a dermoid cyst within the right cerebellar hemisphere. On admission, the patient was neurologically intact. Eye examination revealed left ocular convergent strabismus. Cerebellar signs were negative, and GTR was achieved. The cyst wall was not resected intraoperatively because it was adherent to the sigmoid sinus. No surgery-related complications occurred. The patient was discharged home neurologically intact. Pathological examination revealed a dermoid cyst. The patient was followed up for 12 months without radiological signs of tumor recurrence and remained asymptomatic. 

### 3.10. Case 7

A 17-year-old female patient underwent brain imaging due to behavior changes. The patient was severely disabled as a result of inborn encephalopathy of uncertain origin. Since birth, the patient presented with profound intellectual disability with spastic tetraparesis. Brain imaging was undertaken due to her history of overnight episodes of anxiety involving screaming and hitting her head against the bed. A subsequent brain MRI scan revealed a space-occupying mass lesion, presumably originating from the choroid plexus within the collateral triangle of the lateral ventricle on the right side. On admission, the patient presented with a profound psycho-motor impediment, with severe spastic tetraparesis severely affecting motor coordination. GTR of the tumor was achieved. Postoperatively, the patient presented exacerbation of the left upper limb paresis and fever. Due to the concurrent signs of infection in the blood test, antibiotics were administered. As a result, the signs of infection disappeared, and the left upper limb paresis improved. The patient was discharged home in a neurological condition with a similar severity to that at admission. Pathological examination revealed atypical neurocytoma (WHO grade 2 tumor). At the 45th month of follow-up, tumor recurrence was observed. Since the lesion tended to increase, the patient qualified for reoperation. GTR was achieved. However, following the surgery, the wound dehiscence occurred with cerebrospinal fluid leakage from the surgical wound. Subsequently, the wound was surgically managed. The patient was discharged from the hospital in a clinical state similar to that at admission. She was followed up for the next 69 months without radiological signs of recurrence.

## 4. Discussion

The availability and indications for neuroimaging have increased over time. Consequently, the incidence of incidentally found brain tumors in children is expected to rise, demanding the establishment of the safest and most efficient treatment strategy for those patients [16]. Despite ongoing research, the literature on pediatric brain incidentalomas remains sparse [2,5,9,15,17,18,19,20]. Consequently, no consensus has been reached on whether these lesions should be initially surgically removed or followed up with MRI scans repeated on a regular basis. Due to the long life expectancy of the pediatric population, the optimal management of brain incidentalomas causes a significant dilemma, underpinned by the aim of ensuring patients’ high long-term quality of life (QoL). Therefore, it is debated whether surgical intervention is ethically warranted when performed on an asymptomatic patient, creating a risk of iatrogenic deficits and clinical worsening. On the other hand, the conservative approach based on serial MRI examination carries some risk of tumor progression and MT (even though this is relatively rare [6,21,22,23]), leading to the need for challenging surgical tumor resection and a higher risk of postoperative neurological sequelae.

To date, as reported in the literature, the vast majority of pediatric patients (77.2%) were initially managed conservatively, while 22.8% underwent surgery straight after radiological diagnosis [4]. Delayed surgical tumor resection appeared in 9.5% of patients primarily subjected to radiological surveillance [4]. Notably, neither initial surgery nor delayed surgery was associated with postoperative iatrogenic neurological morbidity [5,9,15,17,18,19,20,24]. Despite the limited cohort, our study further supports the safety of initial surgical resection since no patient experienced new neurological deficits following surgery. 

Notably, one patient in our cohort (patient no. 5), who underwent total craniopharyngioma resection via a fronto-temporal craniotomy, showed clinical signs of precocious puberty during the follow-up period. Initially, the patient was asymptomatic, with no evidence of endocrinological disturbances. Precocious puberty has already been reported as a very rare manifestation of craniopharyngioma [25,26,27,28,29,30,31]. The majority of patients diagnosed with craniopharyngioma initially present with headaches, vomiting, visual impairment, and stunted growth [32,33,34]. Endocrinological pathologies can also be found in the vast majority of patients at admission, while typically they do not constitute the principal presenting complaints [27,35]. We hypothesize that precocious puberty in our initially asymptomatic patient might have resulted from craniopharyngioma itself. Consequently, clinical manifestation would appear later after symptoms indicative of lesion growth and subsequent significant mass effect occur. However, one can also connect the postoperative appearance of symptoms indicative of precocious puberty with an iatrogenic injury to the neural structures of the parasellar region. Being aware of the possible iatrogenic sequelae in patient no. 5, we believe that delayed surgery, if performed in symptomatic patients as a result of tumor growth, might also potentially increase the risk of iatrogenic injury and both neurological and endocrinological complications.

It has been stated that GTR should be the goal in pediatric patients with LGGs [36]. Furthermore, GTR of pediatric LGGs is associated with longer progression-free survival [36,37]. It is speculated that early surgery aimed towards GTR is technically more feasible due to the smaller tumor size and better accessibility of the tumor, lowering the risk of surgery-related morbidity and MT. Lesions located within the eloquent areas or those involving deep structures require particular attention [5]. In our study, one patient with left-sided thalamic ganglioglioma underwent subtotal tumor excision without any surgery-related morbidity (Figure 2). We presume that early surgery instead of delayed surgery would increase the EOR, especially in tumors located within the eloquent brain areas, with minimized risk of iatrogenic injury. To date, the value of early versus delayed tumor resection cannot be unequivocally stated, leaving the question of the optimal time to conduct surgery unanswered. However, particular caution is needed in the selection of patients with tumors involving eloquent brain areas for surgical resection since even early surgery can cause significant neurological injury, impacting the patient’s QoL. 

While the safety of the surgical approach has already been reported (100% patients neurologically intact after the surgery [3]), it still carries some potential risk of iatrogenic neurological injury. Considering the expected long lifetime of pediatric patients, the possible complications associated with surgical resection have to be considered and discussed by the multidisciplinary team and with the patient’s caregivers, as complications can significantly affect the patient’s QoL. The possible complications associated with surgical resection influenced the tendency towards a conservative approach seen in the literature, with 77.2% of patients being initially observed due to a non-suspicious radiological tumor appearance. The trend towards a conservative approach results from the characteristics of LGGs in the pediatric population. Compared to adults, pediatric LGGs present a more benign course, slower growth rate, and low risk of MT, partly justifying the primarily applied conservative approach [3,7]. The MT of LGG is observed in 50–90% of adult patients [22], while MT appears in up to 10% of pediatric LGGs [21,23]. Although relatively rare, MT is associated with a poor prognosis.

Therefore, patients in whom initial surgery is not considered should be closely controlled via serial MRI scans and reconsidered for surgery in the event of radiographic signs of progression/malignancy or symptom appearance. It has been suggested that surgery should initially be performed in cases of mass effect, nodular contrast enhancement, edema, significant tumor growth, or the appearance of neurological symptoms [5,9,15,17,18,19,20,24]. However, there are no definitive radiological criteria for differentiating low-grade from high-grade lesions [4]. Thus, high-grade tumors or MT can be accidentally overlooked, thereafter being associated with fatal consequences. The increasing number of reports indicating the possibility of LGG MT undermines the overall safety of conservative management [4]. 

Although progression-free survival has been estimated at 73% following 30 months of active radiological surveillance, it is still unknown how many patients would require delayed surgery after a longer follow-up period [3]. Since spontaneous tumor regression has been reported in 5% of incidentally found brain tumors thus far, it is unlikely that progression-free survival would stabilize at 73% for many years. Moreover, radiological progression, which forced shifting the management strategy towards immediate surgical intervention, was observed in as many as 22% of initially observed patients [3]. Kozyrev et al. reported on the longest in the literature, a56.5 months-long follow-up involving conservatively managed pediatric patients [5]. The authors reported that 21.3% of conservatively treated patients underwent radiological progression within the mean time of 11.4 months and qualified for surgery. While the literature lacks longer follow-up, the further course of incidentally found brain tumors in children is unknown. Conversely, no recurrence was found among initially surgically treated patients within the mean follow-up of 68.3 months [3]. Noteworthy, it remains impossible to conclude about the overall survival rate in both conservatively and surgically treated patients based on the limited follow-up data reported in the literature. It would be of great interest to perform a multicenter prospective study with long-term follow-up of conservatively managed patients to further define the progression rate, especially during adulthood. We hypothesize that a well-designed prospective study with long-term follow-up would greatly contribute to tip the scales to one approach. 

Furthermore, apart from purely surgical concerns regarding the “to treat or not to treat” dilemma, the radiological follow-up of an incidentally found brain tumor in a child leaves the patient’s caregivers with a significant psychological burden. Therefore, some parents might opt for surgery, despite the lack of radiological or clinical warning criteria, to relieve the psychological burden of having a child with a brain tumor.

While no consensus can be achieved on the basis of existing data and since radiological surveillance and early resection have both advantages and disadvantages, an individualized strategy should be applied to every patient. In addition, clinicians should bear in mind that the active participation of the patient’s family is mandatory in the decision-making process.

### Limitations

The main limitations of our study include its retrospective character and relatively small patient sample. In addition, the lack of a comparison with patients undergoing imaging surveillance precluded any conclusions on the superiority of one particular approach over another.

## 5. Conclusions

The majority of pediatric incidentally found brain tumors are histologically benign. Surgical resection remains a safe therapeutic approach associated with favorable long-term outcomes. Considering the long expected lifetime of pediatric patients, surgical tumor resection can be perceived as an initial approach. Early resection increases the possibility of GTR with a minimized risk of iatrogenic injury. Early resection also eliminates the risk of MT and alleviates the psychological burden for the patient and caregivers.

## Figures and Tables

**Table 1:** Characteristics of patients with incidentally found brain tumors.

Patient No.	Age at Diagnosis	Sex	Reason for Neuroimaging	Location	Histopathology	Extent of Resection (EOR)	Postoperative Clinical State	Follow-Up Clinical Course	Follow-Up Tumor Appearance	Follow-Up Time (Months)
**1**	14 years	F	paranasal sinuses control in pertussis	thalamus	ganglioglioma	subtotal resection	No deficits, asymptomatic	Periodic headaches	No recurrence	111
**2**	11 years	M	shunt control	frontal lobe	pilocytic astrocytoma	total resection	No deficits, asymptomatic	No deficits, asymptomatic	No recurrence	74
**3**	15 years	M	head trauma	temporal lobe	pilocytic astrocytoma	total resection	No deficits, asymptomatic	No deficits, asymptomatic	No recurrence	92
**4**	21 months	M	preterm birth	lateral ventricle	choroid plexus papilloma	total resection	No deficits, vomiting	Periodic syncope	No recurrence	86
**5**	3 years	F	speech development delay	suprasellar region	craniopharyngioma	total resection	No deficits, asymptomatic	Preterm maturation	No regrowth	65
**6**	6 years	F	speech development delay	cerebellum	dermoid cyst	subtotal resection	No deficits, asymptomatic	No deficits, asymptomatic	No recurrence	12
**7**	17 years	F	behavior changes	lateral ventricle	atypical neurocytoma	total resection	No deficits, asymptomatic	No deficits, asymptomatic	Recurrence after 45 months	114

## Data Availability

The data generated during this study are available within the article. Data sets analyzed during the preparation of the current study are available from the corresponding authors upon reasonable request.
